# The Brain’s Response to the Human Voice Depends on the Incidence of Autistic Traits in the General Population

**DOI:** 10.1371/journal.pone.0080126

**Published:** 2013-11-20

**Authors:** Yuko Yoshimura, Mitsuru Kikuchi, Sanae Ueno, Eiichi Okumura, Hirotoshi Hiraishi, Chiaki Hasegawa, Gerard B. Remijn, Kiyomi Shitamichi, Toshio Munesue, Tsunehisa Tsubokawa, Haruhiro Higashida, Yoshio Minabe

**Affiliations:** 1 Research Center for Child Mental Development, Kanazawa University, Kanazawa, Japan; 2 Department of Psychiatry and Neurobiology, Graduate School of Medical Science, Kanazawa University, Kanazawa, Japan; 3 Department of MEG, Yokogawa Electric Corporation, Tokyo, Japan; 4 International Education Center, Kyushu University, Fukuoka, Japan; 5 Department of Anesthesiology, Graduate School of Medical Science, Kanazawa University, Kanazawa, Japan; Tokyo Metropolitan Institute of Medical Science, Japan

## Abstract

Optimal brain sensitivity to the fundamental frequency (F0) contour changes in the human voice is important for understanding a speaker’s intonation, and consequently, the speaker’s attitude. However, whether sensitivity in the brain’s response to a human voice F0 contour change varies with an interaction between an individual’s traits (i.e., autistic traits) and a human voice element (i.e., presence or absence of communicative action such as calling) has not been investigated. In the present study, we investigated the neural processes involved in the perception of F0 contour changes in the Japanese monosyllables “ne” and “nu.” “Ne” is an interjection that means “hi” or “hey” in English; pronunciation of “ne” with a high falling F0 contour is used when the speaker wants to attract a listener’s attention (i.e., social intonation). Meanwhile, the Japanese concrete noun “nu” has no communicative meaning. We applied an adaptive spatial filtering method to the neuromagnetic time course recorded by whole-head magnetoencephalography (MEG) and estimated the spatiotemporal frequency dynamics of event-related cerebral oscillatory changes in beta band during the oddball paradigm. During the perception of the F0 contour change when “ne” was presented, there was event-related de-synchronization (ERD) in the right temporal lobe. In contrast, during the perception of the F0 contour change when “nu” was presented, ERD occurred in the left temporal lobe and in the bilateral occipital lobes. ERD that occurred during the social stimulus “ne” in the right hemisphere was significantly correlated with a greater number of autistic traits measured according to the Autism Spectrum Quotient (AQ), suggesting that the differences in human voice processing are associated with higher autistic traits, even in non-clinical subjects.

## Introduction

Recent cognitive theories of autism spectrum disorder (ASD) have emphasized the importance of considering not only the cognitive and social deficits of individuals with ASD but also the aspects of the intact or even enhanced cognitive abilities [1]–[5]. The over-functioning of brain regions typically involved in primary perceptual functions was recently considered an autistic perceptual endophenotype, and “the Enhanced Perceptual Functioning (EPF) model” [2], [6] has been proposed to explain the superiority of the perceptual flow of information (e.g., enhanced low-level discrimination) in ASD individuals. This model is supported by previous neurophysiological studies demonstrating hypersensitivity for auditory change detection of low-level auditory stimuli in subjects with Asperger syndrome (i.e., an autism spectrum condition), reflected in the enhanced mismatch negativity (MMN) for deviant sounds [7], [8].

The social deficits characteristic of autism spectrum disorders have been observed at lower levels on a continuum throughout the population [9]–[12]. A twin study demonstrated that autistic traits are continuously distributed and moderately to highly heritable in the general population, suggesting that these observations might be arbitrary where cutoffs are made between research designations of “affected” vs. “unaffected” with ASD [10]. This continuum was revealed using tools such as the Social Responsiveness Scale (SRS) [13] or the Autism Spectrum Quotient (AQ) [9]. Intriguingly, a recent study demonstrated that non-clinical subjects with high AQ scores (i.e., higher autistic traits) also show this superiority in the perceptual flow of auditory information [14]. These authors observed that higher AQ scores were significantly correlated with pitch identification scores in non-clinical subjects, and AQ scores were significantly higher in subjects of absolute pitch than in subjects of non-absolute pitch [14]. These results suggest that the differences in cognitive processing are associated with non-clinical subjects who have higher autistic traits and subjects with ASD.

In addition, another recent study has suggested that differences in cognitive processing vary with the complexity of the auditory stimuli in clinical subjects with ASD [15]. Thus, these authors concluded that although individuals with ASD are often more proficient at processing simple, low-level auditory stimuli (e.g., pitch discrimination), they exhibit impaired performance as stimuli become more social and/or as task demands increase (e.g., comprehension, irony and prosodic perception); this pattern has been observed at both behavioral and neural levels [15]–[25]. However, there are no studies concerning the influence of autistic traits on the brain response to socially relevant auditory stimuli (i.e., such as “hi”) in non-clinical subjects.

The precise perception of fundamental frequency (F0) contour changes in human speech is linguistically important, resulting in better intonational (non-lexical) comprehension in both tonal (e.g., Chinese) and non-tonal languages (e.g., English and Japanese). F0 contour changes in Japanese monosyllabic words, rather than carrying a lexical load, usually reflect the speaker’s attitude (e.g., an attention-seeking, emotional, declarative, or interrogative intonation). One of the most used interjections in Japanese is the syllable “ne,” meaning “hi,” “hey,” “look,” or “you know” in English. The monosyllabic word “ne” is frequently used in daily conversation from childhood to adulthood in Japan, either as the final syllable in a sentence or uttered in isolation with a typical high falling F0 pattern to attract the listeners’ attention [26]–[29].

Using magnetoencephalography (MEG), which has a high time resolution, we recently demonstrated significant brain activation (i.e., beta band event related de-synchronization; ERD) in the right temporal area in time windows ranging from 100–300 ms and from 300–500 ms after the onset of “ne” with a typical high falling F0 pattern in healthy subjects [30]. However, research on the semantic processing of language has shown that in healthy subjects, ERD in the beta band consistently reflects left-lateralized brain processing during tasks involving semantic comprehension [31]–[33]. The results from previous studies using ERD analysis in the beta band support the functional hypothesis for brain lateralization, which states that F0 contour changes are lateralized to different hemispheres of the brain based on their functions; i.e., lexical F0 patterns are lateralized to the left hemisphere, whereas intonation patterns signaling speakers’ attitudes are lateralized to the right hemisphere [34].

**Figure 1:**
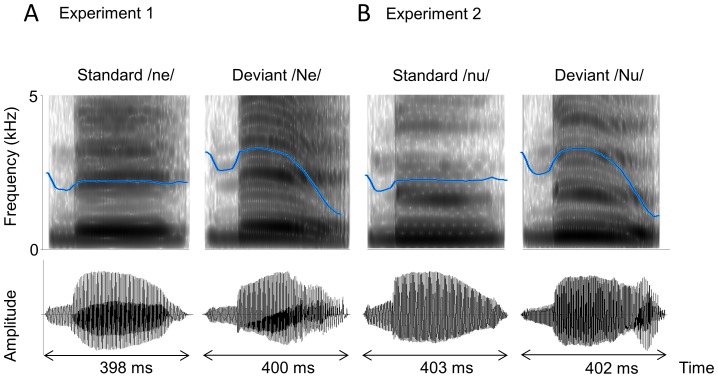
Spectrogram (top) and oscillogram (bottom) of the standard/ne/(A, left) and/nu/(B, left) and the deviant/Ne/(A, right) and/Nu/(B, right) syllables. The blue line in the spectrogram indicates the average pitch contour. Note the near-flat contour in the standard/ne/and/nu/and the high falling pitch contour in the deviant/Ne/and/Nu/. The F0 contours in/ne/and/nu/or/Ne/and/Nu/are almost the same.

However, whether the sensitivity of the brain’s response (i.e., beta band ERD) to human voice F0 contour change would vary as an interaction between a human voice feature (i.e., presence or absence of attention-seeking) and an individual’s traits (i.e., autistic traits) has not previously been investigated. If the listener has higher autistic traits, then the human voice might represent less meaningful stimuli, even when the voice has a communicative intonation. Therefore, in the present study, we hypothesized that non-clinical subjects with higher autistic traits exhibit lower brain responses to human voice stimuli that typically attract the listener’s attention (i.e., presence of attention-seeking) than subjects with lower autistic traits. To investigate this hypothesis, we performed two experimental sessions using two types of human voice monosyllables, “ne” and “nu.” We employed monosyllable sounds with short durations (i.e., approximately 400 ms) to effectively utilize MEG data at a high time resolution. If we use two- or three-syllable words as stimulus sounds, then the duration of the stimuli must be 800–1000 ms, and brain responses to the first-, second- and third-syllable sounds would become contaminated with brain responses to the other syllable sounds. Thus, it would be difficult to identify which specific syllable induced the power change at a specific time and in a specific brain region, thereby losing the advantage of MEG analysis (i.e., a high time resolution). Furthermore, both “ne” and “nu” start with the same consonant ‘n’ (i.e., the alveolar nasal sound), which enables us to generate auditory stimuli with nearly the same F0 contours from the onset of the syllable sounds (Figure 1). For Japanese speakers (at least in Kanazawa city), the Japanese syllable “ne,” which was used in our previous study, attracts the attention of the listener with a typical high falling F0 pattern. However, the Japanese syllable “nu,” an infrequent word used to describe an animal of the cattle family Bovidae (gnu) native to Africa, shows a high falling F0 pattern that has no additional meaning and only reinforces the lexical meaning (i.e., “nu” has no communicative action). Consistent with our previous study, we used an oddball paradigm to assess the spatiotemporal characteristics of beta band ERD that underlies the discrimination of an intonational F0 contour change in the syllables “ne” or “nu.” The MEG analysis of ERD can be used to evaluate stimuli-induced changes in oscillatory brain activity, which might be critically diminished during the trial-averaging process, yielding the conventional mismatch field [35]–[37]. In the present study, we hypothesized that having more autistic traits is associated with increased brain sensitivity to human voice F0 contour changes in the syllable “nu” (the meaningless lower social stimulus with an F0 contour change) but not in the syllable “ne” (the higher social stimulus with communicative action).

## Materials and Methods

### Participants

Twenty healthy, native Japanese volunteers with a mean age of 28.9 ± 6.1 years (SD) (ranging from 23 to 44 years; 12 men and 8 women) participated in this study. All participants were right-handed and had normal hearing and no history of neurological or psychiatric diseases. Handedness was determined based on preference when handling a pen. Subjects were screened using a structured clinical interview for DSM-IV (SCID-I/NP) [38] to exclude a personal history of psychiatric illness. In the present study, six of the 20 participants overlapped with our previous study [30]. All subjects agreed to participate in the study with full knowledge of the experimental nature of the research. Written informed consent was obtained before the start of the experiment, and the Ethics Committee of Kanazawa University Hospital approved the methods and procedures that were used.

### Traits of Autism Spectrum Disorder in the General Population

The traits of ASD have been characterized using the AQ [9]. The test consists of 50 statements, each of which is in a forced choice format. AQ is a valuable tool for rapidly quantifying where on the continuum from autism to normality an individual of average intelligence is situated [9]. In the present study, the mean AQ score was 16.0±5.6 (±1SD).

### Stimuli and Procedures

All participants underwent MEG examination while listening to auditory stimuli presented in an oddball paradigm. In experiment 1, the stimuli consisted of the Japanese monosyllabic sound “ne” pronounced in two different ways (Figure 1A). A repetitive series of utterances of “ne” pronounced with a flat F0 contour (/ne/), was used as a standard. This stimulus carried meaningless intonational information. As a deviant stimulus, we used “ne” pronounced with a high falling F0 contour (/Ne/), which carries intonational information to attract the listeners’ attention. In experiment 2, the stimuli consisted of the meaningless monosyllabic sound “nu” (Figure 1B). A repetitive series of utterances of “nu” pronounced with a flat F0 contour (/nu/), was used as a standard. As a deviant stimulus, we used “nu” pronounced with a high falling F0 contour (/Nu/), which carries meaningless intonational information.

In experiments 1 and 2, the standard and deviant stimuli were randomly presented to the participants at rates of 83% and 17%, respectively. The total number of stimuli presented was 546 (456 standards and 90 deviants). The/ne/and/Ne/(or/nu/and/Nu/) sounds were produced by a female native Japanese speaker and were recorded for stimulus presentation using a condenser microphone (Rode NT1-A) onto a personal computer. In experiment 1, each standard stimulus lasted 398 ms and the duration of the consonant/n/was 78 ms; these durations were 400 and 92 ms, respectively, in the deviant stimuli (Figure 1A). In experiment 2, the standard stimulus lasted 403 ms and the duration of the consonant/n/was 84 ms; these durations were 402 and 91 ms, respectively, in the deviant stimuli (Figure 1B). In experiment 1 and 2, the stimuli were presented with an interstimulus interval (ISI) of 761 and 758 ms, respectively. Stimuli were presented at an average sound pressure level of 65 dB (A-weighted, fast-peak) at the participant’s position, as measured with an integrating sound level meter (Yokogawa LY20). The stimuli were presented binaurally to the participant through a hole in the MEG-chamber by speakers (Harman Kardon, HK195) placed outside the shielded room. Stimuli were presented for 12 minutes in each experiment. To diminish the order effect, 10 subjects started with experiment 1 and ended with experiment 2; the other 10 subject with experiment 2 and ended with experiment 1.

**Figure 2:**
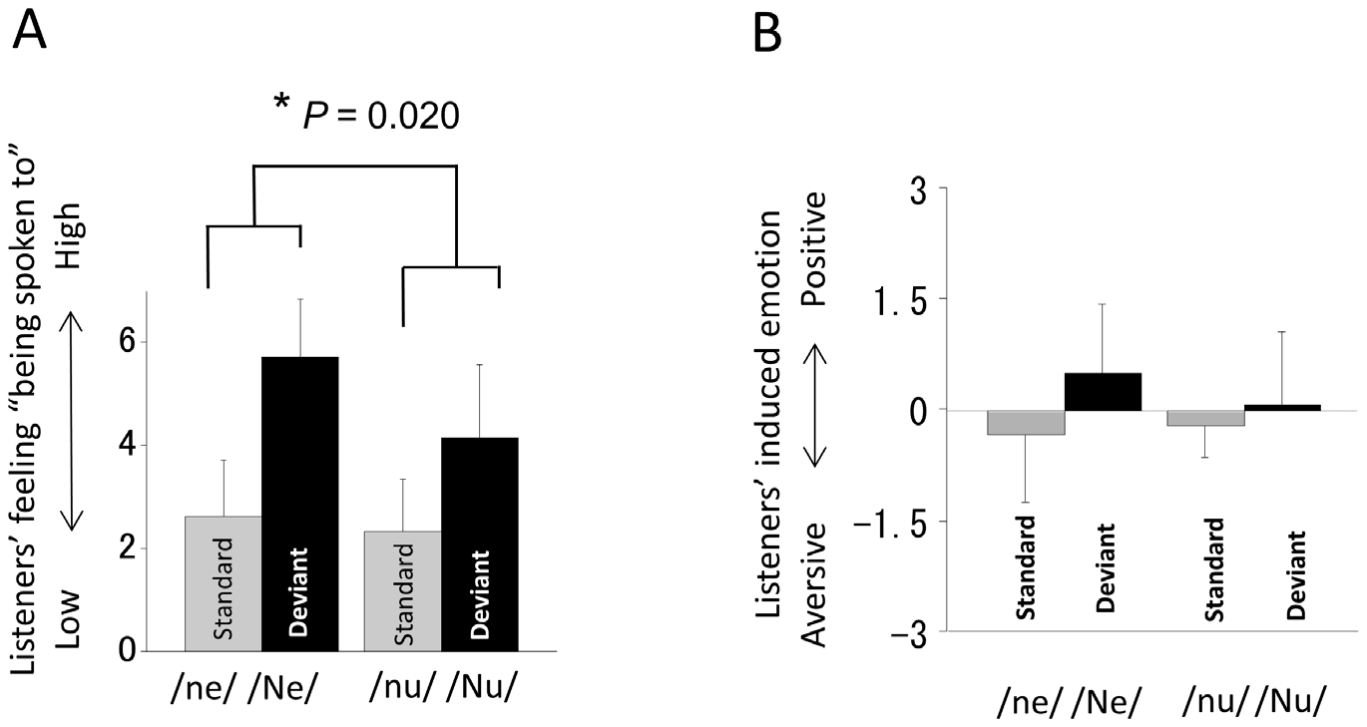
Differences in the intonational meaning of the standard/ne/and the deviant/Ne/or of the standard/nu/and the deviant/Nu/. A, Ratings for listeners’ feelings of “being spoken to” following the standard/ne/or/nu/and the deviant/Ne/or/Nu/utterances; validation was by the subjects in the present study (*n* = 18). Repeated-measures ANOVA demonstrated a significant interaction between two factors (type of F0 contour×type of syllable; F = 6.61, * *P* = 0.020). The F0 contour effect of “ne” was stronger than that of “nu” for this kind of listeners’ feelings. B, Ratings for the emotion (aversive or comfortable) that listeners felt following the standard/ne/or/nu/and the deviant/Ne/or/Nu/utterances; validation is by the subjects in the present study (*n* = 18). There was no significant interaction between two factors (type of F0 contour×type of syllable; F = 2.59, *P* > 0.05), which means that the F0 contour effect of “ne” was not significantly different from that of “nu” for listener’s emotion. Error bars indicate 1 standard deviation.

To validate the differences in the intonational meaning of the standard/ne/and the deviant/Ne/or of the standard/nu/and the deviant/Nu/, 18 of the 20 subjects were asked to evaluate the stimuli on a 7-point rating scale after the MEG recording (2 of the 20 subjects did not participate in this questionnaire because of our lack of preparation). The listeners rated their impression of “being spoken to” and the emotion (aversive or comfortable) they felt. A repeated-measures two-way ANOVA was performed (type of F0 contour×type of syllable) on the rated impressions of each listener. Both factors are intra-subject variables (type of F0 contour and type of syllable). The listener’s feeling of “being spoken to” showed a significant interaction between the two factors (type of F0 contour×type of syllable; F = 6.61, *P* = 0.020; Figure 2A), indicating the F0 contour effect for “ne” was stronger than that for “nu.” However, the emotion (aversive or comfortable) the listener felt showed no significant interaction between two factors (type of F0 contour×type of syllable; F = 2.59, *P* > 0.05; Figure 2B), indicating the F0 contour effect for “ne” was not significantly different from that for “nu” for the listeners’ emotional feelings.

### Measurements

Magnetic fields were measured using a whole-head-type MEG system for adults in a magnetically shielded room (Daido Steel, Nagoya, Japan) at the Laboratory of Yokogawa Electric Corporation in Japan. This system (MEGvision PQA160C; Yokogawa Electric Corporation, Kanazawa, Japan) employs 160 channels of axial-gradiometers, where the coil diameter of the sensors is 15.5 mm and the baseline is 50.0 mm. Band-pass-filtered MEG data (0.16–200 Hz) were collected with the sampling rate of 1000 Hz. The magnetic resonance imaging (MRI) measurements were obtained using a Sigma Excite HD 1.5 T system (GE Yokogawa). All subjects underwent T1-weighted MRI with spherical lipid markers placed at 5 MEG fiduciary points to facilitate the superposition of the MEG coordinate system on the MRI. The MRI comprised 166 sequential slices of 1.2 mm with a resolution of 512×512 points in a field of view of 261×261 mm.

### Data Analysis: Analysis Sequence

First, based on the results of our recent study [30], a single frequency band of interest, beta (13–30 Hz), was isolated from the magnetic field data using band-pass filter for each subject under each stimulus condition. Then, the current density for each voxel was calculated by adaptive spatial filtering using a single spherical volume conductor model that was based on individual MR images. Power changes in the current density between the active and baseline periods were calculated for each voxel with a 5-mm grid spacing. In the present study, we calculated the stimuli-induced power changes containing both phase-locked and non-phase-locked oscillatory power changes. The baseline period was defined as between 200 and 0 ms before the stimulus onset, and the active periods of interest were defined as continuously moving, 200-ms windows from 100–300 ms and 400–600 ms after the stimulus onset. The windows were moved in steps of 100 ms. To equalize the number of epochs for the two conditions (e.g.,/ne/and/Ne/) in each participant, we randomly thinned out the number of epochs for the standard/ne/or/nu/stimuli. Finally, group analysis using Statistical Parametric Mapping (SPM) (Wellcome Department of Cognitive Neurology, London, UK, http://www.fil.ion.ucl.ac.uk/spm/software/spm8/) identified statistically significant differences between the two conditions (i.e.,/ne/and/Ne/or/nu/and/Nu/) for each voxel with a 5-mm side, and these results were visualized as three-dimensional images.

### Data Analysis: Source Reconstruction using Adaptive Spatial Filtering

Adaptive spatial filtering is a spatial filtering approach to source reconstruction that can estimate neuromagnetic activity with high spatial resolution. It does so by forming a linear combination of sensors that can suppress the signals from environmental noise or other brain areas without attenuating the power from the target voxel. The approach is optimized for time–frequency source reconstruction from MEG/EEG data [39], [40]. The adaptive spatial filtering method that we employed is a normalized version of a Van Veen (1997) beamformer [41]. We have created our own software. We defined the magnetic field measured by the *m*-th sensor at time *t* as 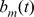, and a set of measured data as a column vector 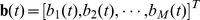 where 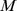 is the total number of sensors and superscript 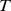 indicates the matrix transpose. The covariance matrix of the measurement was denoted 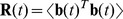 where 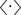 indicates the ensemble average over trials. We assumed that the sensor data arise from elemental dipoles at each spatial location 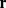, represented by a three-dimensional vector such that 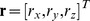. The orientation of each source was defined as the vector 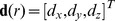, where 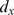, 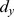, and 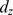 are the angles between the moment vector of the source and the *x-*, *y-*, and *z*-axes, respectively. We defined 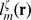 as the output of the *m*-th sensor that would be induced by a unit-magnitude source located at 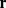 and pointing in the 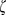 direction. The column vector 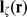 was defined as 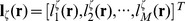. The lead field matrix, which represents the sensitivity of the whole sensor array at 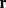, was defined as 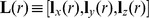. The lead field vector for a unit-dipole oriented in the direction 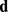 was defined as 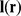, where 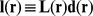.

An adaptive spatial filter [41], [42] estimate of the source moment is given by 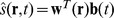, where 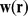 is the weight vector. The weight vector 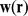 of an adaptive spatial filter called the array-gain constraint minimum variance scalar beamformer is calculated by minimizing 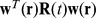 subject to 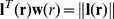. The solution is known to be [43]:
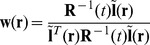
where 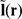 is the normalized lead-field vector defined as 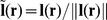. An optimal orientation 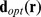 was determined by computing the solution that maximizes output SNR with respect to 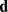 [44], [45].

On the biomagnetic measurements, sometimes is 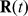 close to a singular matrix. Thus, the errors generated when calculating 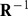 can be reduced by applying a technique called the diagonal loading (Tikhonov regularization) in the field of sensor array processing [46], [47]. This technique calculates 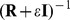, instead of directly calculating 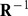. Here 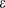 is a controllable parameter and we can recover the output SNR to some extent. We can derive the array-gain-constraint version of the diagonal loading spatial filter whose weight vector is expressed as
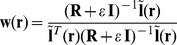



The estimated source power 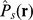 can be computed from the weights 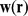 and the covariance 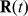: 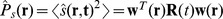.

With a dual-state paradigm, such as those involving power changes on oscillatory activities, one is interested in the change in power from a baseline time period to an active time period. These periods are denoted as vectors of time samples, 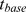 and 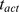, respectively. In this case:
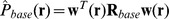


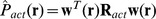
where 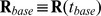 is the covariance of the baseline period and 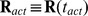 is the covariance of the active period. To improve numerical stability, 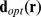 and 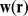 are computed using the average covariance of the active and baseline period, i.e., substituting 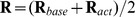. Note that 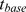 must be the same length as 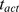.

The contrast between 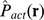 and 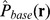 can then be expressed as an *F-ratio* [dB] [40]:
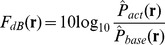



Basic brain rhythms, event-related de-synchronization (ERD), change their signal power due to brain activation. ERD are decreases in oscillatory power and are denoted as negative *F-ratio* values.

### Data Analysis: Group Analysis

The significance of activation across the participants was tested with SPM8. All images were normalized to the MNI space. Then, for each subject at each time window, subtracted images were made by subtracting the images that corresponded to the standard stimuli (i.e.,/ne/or/nu/) from the images corresponded to the deviant stimuli (i.e.,/Ne/or/Nu/). To demonstrate significant beta band ERD during deviant stimuli compared with standard stimuli, one sample *t*-test was performed on the subtracted images for each type of syllable at each time window. Voxels with differences at *P *< 0.001 (uncorrected) were considered statistically significant. Voxel-based correlations were computed between subtracted images (deviant - standard stimuli) and AQ score for each time window using a multiple regression model with the statistical threshold set at *P*< 0.001 uncorrected. In Tables 1–3, Montreal Neurological Institute coordinates in SPM8 were transformed into Talairach coordinates using a non-linear transform of MNI to Talairach (http://imaging.mrc-cbu.cam.ac.uk/imaging/MniTalairach).

**Table 1:** Brain regions with significant differences in beta band ERD between the deviant and standard syllable conditions in experiment 1 (/Ne/−/ne/) and experiment 2 (/Nu/−/nu/).

Time window (ms)	Brain Area Lobe	Anatomical Area	Side	BA	Coordinate†			t Value	Cluster size
					x	y	z		
**/Ne/−/ne/**									
100–300	−	−	−	−	−	−	−	−	
200–400	−	−	−	−	−	−	−	−	
300–500	Temporal Lobe	Inferior Temporal Gyrus	R	20	62.56	−39.21	−15.49	4.57	4
	Temporal Lobe	Superior Temporal Gyrus	R	22	53.76	−48.91	10.41	3.72	1
400–600	Temporal Lobe	Middle Temporal Gyrus	R	21	66.96	−48.91	1.61	4.18	4
	Temporal Lobe	Superior Temporal Gyrus	R	41	40.56	−39.21	10.91	3.99	6
	Occipital Lobe	Lingual Gyrus	R	18	27.36	−68.31	0.61	3.90	2
**/Nu/−/nu/**									
100–300	−	−	−	−	−	−	−	−	
200–400	Occipital Lobe	Lingual Gyrus	R	19	14.16	−53.76	1.36	3.63	2
300–500	Temporal Lobe	Middle Temporal Gyrus	L	21	−69.44	−14.96	−9.84	5.01	18 *
	Occipital Lobe	Lingual Gyrus	L	17	0.96	−92.56	−0.64	4.63	13
400–600	Occipital Lobe	Lingual Gyrus	R	18	9.76	−63.46	5.26	4.38	10
	Occipital Lobe	Cuneus	R	23	14.16	−73.16	9.16	4.12	2
	Temporal Lobe	Middle Temporal Gyrus	L	21	−65.04	−14.96	−5.44	3.82	3
	Occipital Lobe	Lingual Gyrus	L	18	−21.04	−102.26	−9.94	3.66	1
	Temporal Lobe	Inferior Temporal Gyrus	R	20	62.56	−48.91	−11.59	3.58	1

Voxels where statistical significance was found at an individual voxel level of *P* < 0.001 (uncorrected) were presented. Montreal Neurological Institute coordinates in SPM8 were transformed into Talairach coordinates.

† Talairach and Tournoux brain atlas coordinates: x = distance in millimeters to the right (+) or left (–) side of the midline; y = distance anterior (+) or posterior (–) to the anterior commissure; z = distance superior (+) or inferior (–) to a horizontal plane through the anterior and posterior commissures.

BA, Brodmann area; G, Gyrus; R, right; L, left.

* Cluster size, which reached significance at *P*<0.10 (FWE-corrected).

**Table 2:** Brain regions with significant positive correlation with AQ score and beta band ERD in experiment 1 (/Ne/−/ne/) and experiment 2 (/Nu/−/nu/).

Time window (ms)	Brain Area Lobe	Anatomical Area	Side	BA	Coordinate†			t Value	Cluster size
					x	y	z		
**/Ne/−/ne/**									
100–300	Temporal Lobe	Superior Temporal Gyrus	R	22	44.96	−24.66	−1.54	4.20	3
	Temporal Lobe	Middle Temporal Gyrus	R	21	53.76	−29.51	−1.79	3.70	1
200–400	Temporal Lobe	Superior Temporal Gyrus	R	21	49.36	−24.66	−1.54	3.91	1
300–500	Temporal Lobe	Superior Temporal Gyrus	R	22	49.36	−19.81	−5.69	7.74	77 **
400–600	Temporal Lobe	Superior Temporal Gyrus	R	21	49.36	−24.66	−5.94	4.47	15
**/Nu/−/nu/**									
100–300	−	−	−	−	−	−	−	−	
200–400	−	−	−	−	−	−	−	−	
300–500	−	−	−	−	−	−	−	−	
400–600	−	−	−	−	−	−	−	−	

Voxels where statistical significance was found at an individual voxel level of *P* < 0.001 (uncorrected) were presented. Montreal Neurological Institute coordinates in SPM8 were transformed into Talairach coordinates.

† Talairach and Tournoux brain atlas coordinates: x = distance in millimeters to the right (+) or left (–) side of the midline; y = distance anterior (+) or posterior (–) to the anterior commissure; z = distance superior (+) or inferior (–) to a horizontal plane through the anterior and posterior commissures.

BA, Brodmann area; G, Gyrus; R, right; L, left.

** Cluster size, which reached significance at *P*<0.05 (FWE-corrected).

**Table 3:** Brain regions with significant negative correlation with AQ score and beta band ERD in experiment 1 (/Ne/−/ne/) and experiment 2 (/Nu/−/nu/).

Time window (ms)	Brain Area Lobe	Anatomical Area	Side	BA	Coordinate†			t Value	Cluster size
					x	y	z		
**/Ne/−/ne/**									
100–300	−	−	−	−	−	−	−	−	
200–400	−	−	−	−	−	−	−	−	
300–500	−	−	−	−	−	−	−	−	
400–600	Occipital Lobe	Cuneus	L	19	−12.24	−87.71	39.21	3.97	2
**/Nu/−/nu/**									
100–300	Occipital Lobe	Fusiform Gyrus	L	18	−21.04	−97.41	−18.49	4.03	2
	Temporal Lobe	Superior Temporal Gyrus	L	13	−56.24	−39.21	19.71	3.83	1
200–400	Occipital Lobe	Fusiform Gyrus	L	18	−21.04	−92.56	−18.24	4.35	1
300–500	Frontal Lobe	Superior Frontal Gyrus	R	9	44.96	38.39	32.51	3.94	4
	Frontal Lobe	Middle Frontal Gyrus	R	8	40.56	28.69	45.21	3.65	1
400–600	Temporal Lobe	Fusiform Gyrus	R	37	44.96	−53.76	−16.24	4.78	4

Voxels where statistical significance was found at an individual voxel level of *P* < 0.001 (uncorrected) were presented. Montreal Neurological Institute coordinates in SPM8 were transformed into Talairach coordinates.

† Talairach and Tournoux brain atlas coordinates: x = distance in millimeters to the right (+) or left (–) side of the midline; y = distance anterior (+) or posterior (–) to the anterior commissure; z = distance superior (+) or inferior (–) to a horizontal plane through the anterior and posterior commissures.

BA, Brodmann area; G, Gyrus; R, right; L, left.

As a complementary analysis, we also employed a conservative method to control the familywise error (FWE) rate. A command-line tool (3dClusterSim) was used to correct for the inclusion of image-based multiple comparisons [48]. The tool 3dClusterSim is available in the AFNI toolbox (Analysis of Functional NeuroImages, http://afni.nimh.nih.gov/afni/). If we set the statistical threshold for each voxel to a corrected *P*_FWE-corrected_ <0.10, a cluster size of at least 18 voxels was the threshold for significance, based on the results of the Monte Carlo simulation (3dClusterSim using the following parameters: single voxel *P* value = 0.001, 10000 simulations, 16224 voxels (32*38*28 3D grid, 5.0*5.0*5.0 mm^∧^3 voxel sizes) in the spherical mask, and 20-mm FWHM). If we set the statistical threshold for each voxel to a corrected *P*_ FWE-corrected_ <0.05, a cluster size of at least 25 voxels was the threshold for significant, based on the results of the Monte Carlo simulation.

## Results

### Beta Band ERD during Deviant Stimuli Compared with Standard Stimuli for “ne” and “nu”

**Figure 3:**
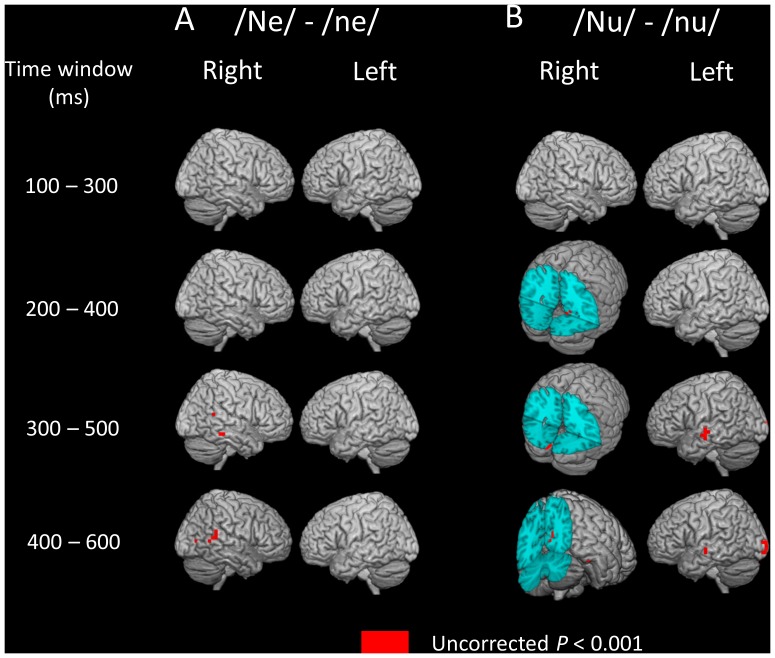
Statistical parametric mapping analyses revealed significant ERD in the beta band during deviant stimuli compared with standard stimuli for the deviant syllables/Ne/(A) and/Nu/(B). A, The F0 contour effect of “ne” induced a significant ERD in the beta band mainly in the right temporal area in two time windows: 300–500 ms and 400–600 ms after stimulus onset. B, The F0 contour effect of “nu” induced a significant ERD in the beta band in three time windows: 200–400 ms (in the right occipital areas), 300–500 ms (in the left temporal and occipital areas), and 400–600 ms (in the bilateral temporal and occipital areas) after stimulus onset.

The results obtained with the “ne” syllable (i.e., an interjection such as “hi”) are shown in Table 1 and Figure 3A. Whole-brain SPM analysis revealed significant ERD in the beta band, associated with the deviant stimulus in two time windows: 300–500 ms (in the right temporal and occipital areas) and 400–600 ms (in the right temporal area) after stimulus onset. The results obtained with the “nu” syllable (i.e., a concrete noun) are shown in Table 1 and Figure 3B. Whole-brain SPM analysis revealed significant ERD in the beta band, associated with the deviant stimulus in three time windows: 200–400 ms (in the right occipital areas), 300–500 ms (in the left temporal and occipital areas), and 400–600 ms (in the bilateral temporal and occipital areas) after stimulus onset.

If we employed a conservative method to control the FWE rate using the cluster size (*P*_FWE-corrected_ <0.10), there was a significant ERD in the beta band associated with the deviant stimulus “nu” during the 300–500 ms time window (in the left temporal area), whereas there were no significant ERDs associated with the deviant stimulus “ne” in any of the time windows. If we employed a more conservative method to control the FWE rate using the cluster size (*P*_FWE-corrected_ <0.05), there were no significant ERDs associated with the deviant stimulus “nu” in any of the time windows.

### Correlation between Beta Band ERD Following “ne” Utterance and AQ Score

**Figure 4:**
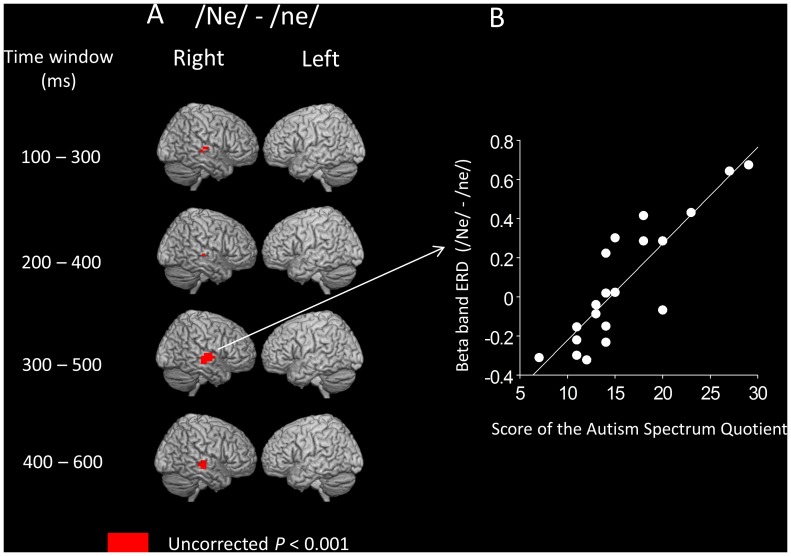
Statistical parametric mapping analyses revealed significant correlation between beta band ERD and AQ score for the “ne” syllable. A, The AQ score is positively correlated with differences in beta band ERD (high falling F0 contour “ne” – flat F0 contour “ne”) in the right superior temporal area in the all-time windows. Note that the region where the significant correlation was observed was located a few centimeters forward of the regions where significant ERD were observed during deviant stimuli compared with the standard stimuli/ne/for the deviant syllables/Ne/(figure 3). B, Scatter plot of the AQ scores and differences in beta band ERD (high falling F0 contour/Ne/− flat F0 contour/ne/) during a time window of 300–500 ms in the right superior temporal area (one voxel) in all participants. Higher values in beta band ERD (/Ne/−/ne/) suggest a prominent decrease in the beta band oscillation after the auditory stimuli/Ne/compared with/ne/.

The results obtained from the whole-brain SPM correlation analysis between beta band ERD and AQ score for the “ne” syllable (i.e., interjection such as “hi”) are shown in Tables 2 and 3 and Figure 4. Significant positive correlations were found in the right temporal area in four time windows: 100–300 ms, 200–400 ms, 300–500 ms, and 400–600 ms after stimulus onset (Table 2). As shown in Figures 3 and 4, the region showing a significant correlation was located a few centimeters forward of the regions where significant ERD were observed during deviant stimuli compared with the standard stimuli/ne/for the deviant syllables/Ne/. Significant negative correlations were found in the occipital area in the 400–600 ms time window (Table 3).

If we employed a conservative method to control the FWE rate using the cluster size (*P*_FWE-corrected_ <0.10), there was a significant positive correlation between the beta band ERD and the AQ score for the “ne” syllable during the 300–500 ms time window (in the right temporal area). Even if we employed a more conservative method to control the FWE rate (*P*_FWE-corrected_ <0.05), this significant positive correlation persisted.

### Correlation between Beta Band ERD Following “nu” Utterance and AQ Score

**Figure 5:**
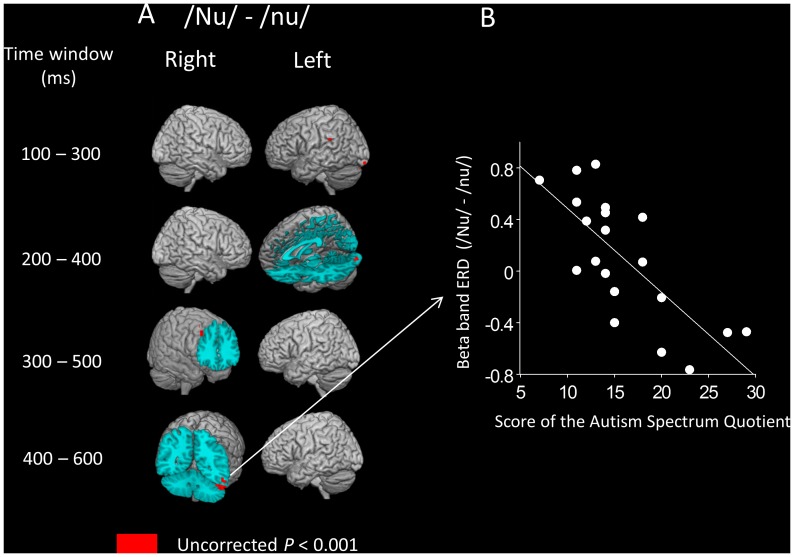
Statistical parametric mapping analyses revealed significant correlation between beta band ERD and AQ score for the “nu” syllable. A, The AQ score is negatively correlated with differences in beta band ERD (high falling F0 contour “nu” – flat F0 contour “nu”) in the four time windows 100–300 ms (in the left temporal and occipital areas), 200–400 ms (in the left occipital areas), 300–500 ms (in the right frontal areas), and 400–600 ms (in the right temporal areas). B, Scatter plot of the AQ scores and differences in beta band ERD (high falling F0 contour/Nu/− flat F0 contour/nu/) during a time window of 400–600 ms in the right fusiform gyrus (one voxel) in all participants. Higher values in beta band ERD (/Nu/−/nu/) suggest a prominent decrease in the beta band oscillation after the auditory stimuli/Nu/compared with/nu/.

The results obtained from the whole-brain SPM correlation analysis between beta band ERD and AQ score for the “nu” syllable (i.e., a concrete noun syllable) are shown in Tables 2 and 3 and Figure 5. There were no significant positive correlations between beta band ERD and AQ score in any time windows (Table 2). There were significant negative correlations in four time windows: 100–300 ms (in the left temporal and occipital areas), 200–400 ms (in the left occipital areas), 300–500 ms (in the right frontal areas), and 400–600 ms (in the right temporal areas) after stimulus onset (Table 3, Figure 5).

If we employed a conservative method to control the FWE rate using the cluster size (*P*_FWE-corrected_ <0.10), there were no significant positive or negative correlations between the beta band ERD and the AQ score for the “nu” syllable in any of the time windows.

## Discussion

### Beta Band ERD in “ne” and “nu”

If we employed a conservative method to control the FWE rate using the cluster size (*P*_FWE-corrected_ <0.05), no significant correlations were observed. Therefore, the following discussions of the human voice-induced ERD in the beta band are at risk for a Type I error.

From the perspective of language lateralization in the human brain, two competing hypotheses (functional and acoustic) have been proposed [34], one of which may explain the neural mechanisms that underlie the perception of F0 changes in “ne” and “nu.” The functional hypothesis states that F0 patterns are lateralized to different hemispheres of the brain based on their functions; i.e., lexical F0 patterns are lateralized to the left hemisphere, whereas intonation patterns, which signal the speakers attitude, are lateralized to the right hemisphere [34]. The acoustic hypothesis states that all F0 (or pitch) patterns are lateralized to the right hemisphere regardless of their function [49]–[51].

In our previous study, we could not determine which hypothesis (i.e., the functional hypothesis or the acoustic hypothesis) could explain our results due to an inadequate experimental design. In the present study, we reconstructed our previous experimental design but used two types of monosyllable to investigate which hypothesis can explain our previous findings. One is the Japanese syllable “ne,” which was used in our previous study [30], and the other is the other Japanese syllable “nu,” which means gnu (a member the family Bovidae). We demonstrated activation of the left middle temporal lobe (i.e., significant ERD in the beta band) for F0 pattern changes with the lexical syllable “nu” (Figure 3B), which was consistent with previous beta band ERD studies that often implicated the left hemisphere in semantic aspects of speech [32], [33]. As shown in Figure 3B, the brain activation in the right hemisphere (i.e., the beta band ERD) that was observed when using the syllable “ne” in our previous study [30] was also replicated in the present study, even though the significance of this effect was not robust.

These results support the functional hypothesis (i.e., intonational F0 contour changes that signal the speaker’s intention are more lateralized to the right hemisphere, whereas those changes that reinforce lexical meaning are more lateralized to the left hemisphere) [34]. Although the precise functional significance of the beta band ERD remains a matter of debate and caution must be exercised when drawing any definitive conclusions based on the results of non-conservative methods (e.g., *P*< 0.001 uncorrected) or based only on variances between 2 monosyllable exemplars, this spatiotemporal sequence might elucidate the dynamic process for intonational F0 contour perception within the right and left hemispheres.

### Correlation between Beta Band ERD in the Temporal Lobes and Autistic Traits

The over-functioning of brain regions typically involved in primary perceptual functions was recently considered as one of the autistic perceptual endophenotypes, and the EPF model was proposed to explain the superiority of the perceptual flow of information in individuals with ASD in comparison with higher-order operations [2]. As previously discussed, individuals with ASD often exhibit enhanced discrimination of low-level acoustic information (i.e., pitch) from pure tones relative to typically developing controls [16]–[20], [52]. However, when more spectrally and temporally complex stimuli such as speech are presented or task demands are increased using more complex stimulus paradigms, individuals with ASD tend to exhibit impaired and/or atypical performance [21]–[25], [53]–[56].

Based on previous studies, we hypothesized that the presence of more autistic traits is associated with a greater brain sensitivity to the human voice F0 contour change in the syllable “nu” (the lower social stimulus that has no meaning associated with the F0 contour change) but not in the syllable “ne” (the higher social stimulus that has an associated meaning of communicative action). Contrary to our hypothesis, we demonstrated that higher activation in the right temporal lobe during the social stimulus “ne” was associated with more autistic traits measured by AQ and no significant positive correlation was found between the brain activation and the prevalence of autistic traits for the non-social stimulus “nu.” The region where brain activation (i.e., prominent ERD) was associated with more autistic traits was located a few centimeters forward of the regions where significant brain activation (i.e., prominent ERD) was observed during deviant stimuli compared with the standard stimuli/ne/for the deviant syllables/Ne/. Even if we employed a conservative method to control the FWE rate using the cluster size (*P*_FWE-corrected_ <0.05), the significance of this effect persisted. These results might reflect functional compensatory mechanisms in which these regions augment brain responses to the human voice, including social information, and therefore, subjects with relatively high AQ scores remain clinically intact. These results might also suggest that there are autistic trait-dependent brain regions, at least in non-clinical subjects, which process higher social human voice information.

The unexpected results for the social stimulus “ne” may be attributed to the fact that we made a wrong assumption based on the previous studies on ASD subjects but not those on non-clinical individual with higher autistic traits. We should have considered the fact that there are differences in linguistic ability between ASD subjects and non-clinical subjects. All the subjects in the present study have no obvious deficits in linguistic ability, whereas the failure to develop sophisticated language (i.e., phonological, semantic, syntactic, pragmatic and prosodic levels) is one of the earliest signs of ASD, although there are a wide variety of symptoms, behaviors, and types of disorders [57], [58]. The various linguistic impairments in ASD may contribute to the impaired and/or atypical brain responses reported in the previous study when more complex human speech paradigms were used, despite the superiority of perceptual flow for low-level information (e.g., pitch detection) in ASD subjects. In the present study, all the subjects had no obvious deficits in any language rules and therefore the superiority of the perceptual flow of information in individuals with more autistic traits was observed, without any unfavorable effects from their linguistic ability.

With respect to the human voice “nu,” if we employed a conservative method to control the FWE rate using the cluster size (*P*_FWE-corrected_ <0.05), no significant correlations were observed. Therefore, the following discussions on the human voice “nu” are at risk for a Type I error. The unexpected results for the non-social stimulus “nu” may be attributed to the fact that human voice “nu” was inadequate stimuli to elicit the right hemispheric brain hypersensitivity to voice F0 contour changes in non-clinical subjects with higher autistic traits. We observed a significant brain response in the left middle temporal lobe during the processing of human voice F0 contour changes for the syllable “nu.” Although “nu” is an infrequent word in Japan, human voice F0 contour changes for “nu” may reinforce the lexical meaning (i.e., the animal’s name, gnu), and may contribute to the observed left middle temporal activation for auditory lexical processing. As the presence of more autistic traits are reflected in brain hypersensitivity in the right temporal area, which has been implicated in Asperger syndrome [8], [59], concrete nouns, such as “nu,” may be inadequate stimuli to elicit the right hemispheric brain hypersensitivity to voice F0 contour changes in non-clinical subjects with higher autistic traits.

Caution must be exercised before drawing any definitive conclusions because we focused on autistic traits only in the healthy population without social or language deficits. Further study with a larger sample that encompasses ASD (including autism, high functioning autism and Asperger syndrome) and various linguistic levels is necessary to investigate whether the sensitivity in the brain’s response to human voice F0 contour changes varies with an interaction between an individual’s heterogeneity of autism symptoms and features of auditory stimuli (i.e., lower to higher level). However, our results suggest that the differences in human voice processing style are associated with higher autistic traits even in non-clinical subjects.

### Correlation between Beta Band ERD in the Occipital Lobes and Autistic Traits

With respect to the occipital lobes, if we employed a conservative method to control the FWE rate using the cluster size (*P*_FWE-corrected_ <0.05), no significant correlations were observed. Therefore, the following discussions of the occipital lobes are at risk for a Type I error. In the present study, an F0 contour change in the concrete noun “nu” induced the beta band ERD in the occipital areas. As beta band ERD have been associated with brain activation in the visual area [60], these results suggest that the brain’s processing of human speech involving concrete words would induce brain activity in the visual association areas (e.g., the fusiform gyri); this interpretation is consistent with previous studies [61], [62]. Although we could not draw any definitive conclusions from the experimental design in the present study, the imageable word “nu” (i.e., forming an image of a gnu) may have contributed to the brain activity in the visual association areas. Unexpectedly, our study demonstrated that prominent ERD in the occipital lobes was associated with a lower incidence of autistic traits. Further study is necessary to elucidate whether the brain’s response in the visual association area to human speech involving concrete words would vary with an interaction between an individual’s traits (i.e., their autistic traits) and features of nouns (i.e., more or less imageable).

### Limitations

There were some limitations to the present study. First, because we did not perform the Wada test, we could not determine the dominant hemisphere in each subject. Using the Wada test, a previous study demonstrated that in 4% of right-handed people and 14% of left-handed people, the right hemisphere is dominant in language and speech function [63]. Although all participants in the present study were right-handed, a case with a right (reversed) hemispheric dominance is possible and would be a robust confounding factor. Second, there were differences of 14 and 7 ms in the consonant duration between the standard and deviant “ne” and “nu” syllables, respectively. Although these differences are less than 1.8% and 0.9% of the respective ISIs and are therefore difficult to perceive, they are a possible confounding effect that could not be avoided. Third, the Japanese language has various dialects, with differences among regions in Japan. In the Kanto region (including Tokyo city, Japan), “ne” is often used as an interjectory particle when attempting to attract the listener’s attention. However, in the Kansai region (including Osaka city, Japan), “na” is often used instead of “ne.” In addition, caution must be exercised when drawing any definitive conclusions based only on variances between 2 monosyllable exemplars. Fourth, although all participants were typically developed and no language problem was spontaneously reported, further study with the precise measurement of language abilities is necessary. Fifth, we focused on autistic traits only in a non-clinical population without social or language deficits. Therefore, we should also consider the fact that the autistic traits measured using the AQ are not uniquely associated with ASD, and higher AQ scores do not always represent the broader ASD phenotype. Further studies using additional methods to identify autistic traits (e.g., the Social Responsiveness Scale for Adults [12]) and larger sample sizes that encompass ASD (including autism, high functioning autism and Asperger syndrome) are necessary to investigate whether the sensitivity in the brain responses to human voice F0 contour changes vary with an interaction between the heterogeneity of the autistic symptoms of the individual and the features of the human voice (i.e., the presence or absence of a communicative action meaning, such as calling).

## Conclusions

We investigated the neurocognitive processes involved in the perception of F0 contour changes in the Japanese monosyllabic words “ne” and “nu” in the general population using MEG with an adaptive spatial filtering method. During the perception of an F0 contour change in “ne,” which attracts the listener’s attention, there was an ERD in beta band oscillation in the right temporal lobe. Unexpectedly, the prominent ERD after social stimuli was significantly correlated with higher AQ scores. During the perception of F0 contour change in “nu,” which is the name of an animal, there was an ERD in beta band oscillation in the left middle temporal lobe. By using a method with high spatiotemporal resolution, from the perspective of language lateralization in the human brain, this study supported the functional hypothesis (i.e., the left hemisphere is used for lexical processing and the right hemisphere is used for prosodic processing). Unexpectedly, our results suggested that the right hemispheric brain hypersensitivity to changes in the human voice that reflect a speaker’s attitude was associated with the presence of more autistic traits in non-clinical subjects. Even if we employed a conservative method to control the FWE rate using the cluster size (*P*_FWE-corrected_ <0.05), the significance of this effect persisted (i.e., the ERD observed during the social stimulus “ne” in the right hemisphere significantly correlated with an increased incidence of autistic traits).

## References

[1] Mottron L (2011) Changing perceptions: The power of autism. Nature 479: 33–35. 22051659 10.1038/479033a

[2] Mottron L, Dawson M, Soulieres I, Hubert B, Burack J (2006) Enhanced perceptual functioning in autism: an update, and eight principles of autistic perception. J Autism Dev Disord 36: 27–43. 16453071 10.1007/s10803-005-0040-7

[3] Samson F, Mottron L, Soulieres I, Zeffiro TA (2011) Enhanced visual functioning in autism: An ALE meta-analysis. Hum Brain Mapp. 10.1002/hbm.21307 PMC6870295 21465627

[4] Perreault A, Gurnsey R, Dawson M, Mottron L, Bertone A (2011) Increased sensitivity to mirror symmetry in autism. PLoS One 6: e19519. 21559337 10.1371/journal.pone.0019519 PMC3084879

[5] Kikuchi M, Yoshimura Y, Shitamichi K, Ueno S, Hirosawa T, et al (2013) A custom magnetoencephalography device reveals brain connectivity and high reading/decoding ability in children with autism. Sci Rep 3: 1139. 23355952 10.1038/srep01139 PMC3555087

[6] Mottron L, Bouvet L, Bonnel A, Samson F, Burack JA, et al (2013) Veridical mapping in the development of exceptional autistic abilities. Neurosci Biobehav Rev 37: 209–228. 23219745 10.1016/j.neubiorev.2012.11.016

[7] Kujala T, Aho E, Lepisto T, Jansson-Verkasalo E, Nieminen-von Wendt T, et al (2007) Atypical pattern of discriminating sound features in adults with Asperger syndrome as reflected by the mismatch negativity. Biol Psychol 75: 109–114. 17257732 10.1016/j.biopsycho.2006.12.007

[8] Lepisto T, Nieminen-von Wendt T, von Wendt L, Naatanen R, Kujala T (2007) Auditory cortical change detection in adults with Asperger syndrome. Neurosci Lett 414: 136–140. 17197079 10.1016/j.neulet.2006.12.009

[9] Baron-Cohen S, Wheelwright S, Skinner R, Martin J, Clubley E (2001) The autism-spectrum quotient (AQ): evidence from Asperger syndrome/high-functioning autism, males and females, scientists and mathematicians. J Autism Dev Disord 31: 5–17. 11439754 10.1023/a:1005653411471

[10] Constantino JN, Todd RD (2003) Autistic traits in the general population: a twin study. Arch Gen Psychiatry 60: 524–530. 12742874 10.1001/archpsyc.60.5.524

[11] Constantino JN (2011) The quantitative nature of autistic social impairment. Pediatr Res 69: 55R–62R. 10.1203/PDR.0b013e318212ec6e PMC3086844 21289537

[12] Constantino JN, Todd RD (2005) Intergenerational transmission of subthreshold autistic traits in the general population. Biol Psychiatry 57: 655–660. 15780853 10.1016/j.biopsych.2004.12.014

[13] Constantino JN (2002) The Social Responsiveness Scale: Los Angeles, Calif. Western Psychological Services.

[14] Dohn A, Garza-Villarreal EA, Heaton P, Vuust P (2012) Do musicians with perfect pitch have more autism traits than musicians without perfect pitch? An empirical study. PLoS One 7: e37961. 22666425 10.1371/journal.pone.0037961 PMC3364198

[15] O’Connor K (2012) Auditory processing in autism spectrum disorder: a review. Neurosci Biobehav Rev 36: 836–854. 22155284 10.1016/j.neubiorev.2011.11.008

[16] Bonnel A, Mottron L, Peretz I, Trudel M, Gallun E, et al (2003) Enhanced pitch sensitivity in individuals with autism: a signal detection analysis. J Cogn Neurosci 15: 226–235. 12676060 10.1162/089892903321208169

[17] Bonnel A, McAdams S, Smith B, Berthiaume C, Bertone A, et al (2010) Enhanced pure-tone pitch discrimination among persons with autism but not Asperger syndrome. Neuropsychologia 48: 2465–2475. 20433857 10.1016/j.neuropsychologia.2010.04.020

[18] Heaton P, Hudry K, Ludlow A, Hill E (2008) Superior discrimination of speech pitch and its relationship to verbal ability in autism spectrum disorders. Cogn Neuropsychol 25: 771–782. 18720290 10.1080/02643290802336277

[19] Heaton P, Williams K, Cummins O, Happe F (2008) Autism and pitch processing splinter skills: a group and subgroup analysis. Autism 12: 203–219. 18308768 10.1177/1362361307085270

[20] Jones CR, Happe F, Baird G, Simonoff E, Marsden AJ, et al (2009) Auditory discrimination and auditory sensory behaviours in autism spectrum disorders. Neuropsychologia 47: 2850–2858. 19545576 10.1016/j.neuropsychologia.2009.06.015

[21] Dawson G, Meltzoff AN, Osterling J, Rinaldi J, Brown E (1998) Children with autism fail to orient to naturally occurring social stimuli. J Autism Dev Disord 28: 479–485. 9932234 10.1023/a:1026043926488

[22] Dawson G, Toth K, Abbott R, Osterling J, Munson J, et al (2004) Early social attention impairments in autism: social orienting, joint attention, and attention to distress. Dev Psychol 40: 271–283. 14979766 10.1037/0012-1649.40.2.271

[23] Gervais H, Belin P, Boddaert N, Leboyer M, Coez A, et al (2004) Abnormal cortical voice processing in autism. Nat Neurosci 7: 801–802. 15258587 10.1038/nn1291

[24] Kuhl PK, Coffey-Corina S, Padden D, Dawson G (2005) Links between social and linguistic processing of speech in preschool children with autism: behavioral and electrophysiological measures. Dev Sci 8: F1–F12. 15647058 10.1111/j.1467-7687.2004.00384.x

[25] Whitehouse AJ, Bishop DV (2008) Do children with autism ‘switch off’ to speech sounds? An investigation using event-related potentials. Dev Sci 11: 516–524. 18576959 10.1111/j.1467-7687.2008.00697.x

[26] Anderson V, Hiramoto M, Wong A (2007) Prosodic Analysis of the Interactional Particle Ne in Japanese Gendered Speech. Japanese/Korean Linguistics 15: 43–54.

[27] Cook HM (1990) The sentence-final particle ne as a tool for cooperation in Japanese convcrsation. Thc Stanford Linguistic Association, Stanford.

[28] Kajikawa S, Amano S, Kondo T (2004) Speech overlap in Japanese mother-child conversations. J Child Lang 31: 215–230. 15053091

[29] Squires T (2009) A discourse Anlysis of the Japanese Particle sa. Pragmatics 4: 1–29.

[30] Ueno S, Okumura E, Remijn GB, Yoshimura Y, Kikuchi M, et al (2012) Spatiotemporal frequency characteristics of cerebral oscillations during the perception of fundamental frequency contour changes in one-syllable intonation. Neurosci Lett 515: 141–146. 22465137 10.1016/j.neulet.2012.03.031

[31] Hirata M, Koreeda S, Sakihara K, Kato A, Yoshimine T, et al (2007) Effects of the emotional connotations in words on the frontal areas–a spatially filtered MEG study. Neuroimage 35: 420–429. 17188899 10.1016/j.neuroimage.2006.11.025

[32] Kim JS, Chung CK (2008) Language lateralization using MEG beta frequency desynchronization during auditory oddball stimulation with one-syllable words. Neuroimage 42: 1499–1507. 18603004 10.1016/j.neuroimage.2008.06.001

[33] Yamamoto M, Ukai S, Shinosaki K, Ishii R, Kawaguchi S, et al (2006) Spatially filtered magnetoencephalographic analysis of cortical oscillatory changes in basic brain rhythms during the Japanese ‘Shiritori’ Word Generation Task. Neuropsychobiology 53: 215–222. 16888404 10.1159/000094835

[34] Wong PC (2002) Hemispheric specialization of linguistic pitch patterns. Brain Res Bull 59: 83–95. 12379438 10.1016/s0361-9230(02)00860-2

[35] Dietl T, Dirlich G, Vogl L, Lechner C, Strian F (1999) Orienting response and frontal midline theta activity: a somatosensory spectral perturbation study. Clin Neurophysiol 110: 1204–1209. 10423186 10.1016/s1388-2457(99)00057-7

[36] Makeig S (1993) Auditory event-related dynamics of the EEG spectrum and effects of exposure to tones. Electroencephalogr Clin Neurophysiol 86: 283–293. 7682932 10.1016/0013-4694(93)90110-h

[37] Wei J, Zhao L, Yan G, Duan R, Li D (1998) The temporal and spatial features of event-related EEG spectral changes in 4 mental conditions. Electroencephalogr Clin Neurophysiol 106: 416–423. 9680154 10.1016/s0013-4694(97)00161-2

[38] First MB, Spitzer RL, Miriam G, Williams JBW (1996) Structured Clinical Interview for DSM-IV-TR Axis I Disorders, Research Version, Patient/Non-patient Edition (SCID-I/P) or (SCID-I/NP). New York: Biometrics Research, New York State Psychiatric Institute November.

[39] Sekihara K, Nagarajan SS (2008) Adaptive Spatial Filters for Electromagnetic Brain Imaging: Springer-Verlag.

[40] Dalal SS, Guggisberg AG, Edwards E, Sekihara K, Findlay AM, et al (2008) Five-dimensional neuroimaging: localization of the time-frequency dynamics of cortical activity. Neuroimage 40: 1686–1700. 18356081 10.1016/j.neuroimage.2008.01.023 PMC2426929

[41] van Veen BD, van Drongelen W, Yuchtman M, Suzuki A (1997) Localization of brain electrical activity via linearly constrained minimum variance, spatial filtering. IEEE Trans Biomed Eng vol. 44: 867–880. 10.1109/10.623056 9282479

[42] van Veen BD, Buckley KM (1988) Beamforming: A versatile approach to spatial filtering. IEEE ASSP Magazine vol. 5: 4–24.

[43] Robinson SE, Vrba J (1999) Functional neuroimaging by synthetic aperture magnetometry. In: Yoshimoto T, Kotani M, Kuriki S, Karibe H, Nakasato N, editors. Recent Advances in Biomagnetism. Sendai: Tohoku University Press.

[44] Sekihara K, Nagarajan SS, Poeppel D, Marantz A (2004) Asymptotic SNR of scalar and vector minimum-variance beamformers for neuromagnetic source reconstruction. IEEE Trans Biomed Eng 51: 1726–1734. 15490820 10.1109/TBME.2004.827926 PMC4041989

[45] Sekihara K, Scholz B (1996) Generalized Wiener estimation of threedimensional current distribution from biomagnetic measurements. In: Aine CJ, editor. Biomag 96: Proceedings of the Tenth International Conference on Biomagnetism: Springer-Verlag. 338–341.

[46] Carlson BD (1988) Covariance matrix estimation errors and diagonal loading in adaptive arrays. IEEE Trans Aerospace and Electronic Systems vol. 24: 397–401.

[47] Cox H, Zeskind RM, Owen MM (1987) Robust adaptive beamforming. IEEE Trans Signal Process vol. 35: 1365–1376.

[48] Cox RW (2012) AFNI: what a long strange trip it’s been. Neuroimage 62: 743–747. 21889996 10.1016/j.neuroimage.2011.08.056 PMC3246532

[49] Ren GQ, Yang Y, Li X (2009) Early cortical processing of linguistic pitch patterns as revealed by the mismatch negativity. Neuroscience 162: 87–95. 19374939 10.1016/j.neuroscience.2009.04.021

[50] Zatorre RJ, Belin P (2001) Spectral and temporal processing in human auditory cortex. Cereb Cortex 11: 946–953. 11549617 10.1093/cercor/11.10.946

[51] Zatorre RJ, Belin P, Penhune VB (2002) Structure and function of auditory cortex: music and speech. Trends Cogn Sci 6: 37–46. 11849614 10.1016/s1364-6613(00)01816-7

[52] O’Riordan M, Passetti F (2006) Discrimination in autism within different sensory modalities. J Autism Dev Disord 36: 665–675. 16639532 10.1007/s10803-006-0106-1

[53] Fujikawa-Brooks S, Isenberg AL, Osann K, Spence MA, Gage NM (2010) The effect of rate stress on the auditory brainstem response in autism: a preliminary report. Int J Audiol 49: 129–140. 20151887 10.3109/14992020903289790

[54] Kallstrand J, Olsson O, Nehlstedt SF, Skold ML, Nielzen S (2010) Abnormal auditory forward masking pattern in the brainstem response of individuals with Asperger syndrome. Neuropsychiatr Dis Treat 6: 289–296. 20628629 10.2147/ndt.s10593 PMC2898167

[55] Lepisto T, Kuitunen A, Sussman E, Saalasti S, Jansson-Verkasalo E, et al (2009) Auditory stream segregation in children with Asperger syndrome. Biol Psychol 82: 301–307. 19751798 10.1016/j.biopsycho.2009.09.004 PMC2771139

[56] Teder-Salejarvi WA, Pierce KL, Courchesne E, Hillyard SA (2005) Auditory spatial localization and attention deficits in autistic adults. Brain Res Cogn Brain Res 23: 221–234. 15820630 10.1016/j.cogbrainres.2004.10.021

[57] De Giacomo A, Fombonne E (1998) Parental recognition of developmental abnormalities in autism. Eur Child Adolesc Psychiatry 7: 131–136. 9826299 10.1007/s007870050058

[58] Wetherby AM, Woods J, Allen L, Cleary J, Dickinson H, et al (2004) Early indicators of autism spectrum disorders in the second year of life. J Autism Dev Disord 34: 473–493. 15628603 10.1007/s10803-004-2544-y

[59] Lepisto T, Silokallio S, Nieminen-von Wendt T, Alku P, Naatanen R, et al (2006) Auditory perception and attention as reflected by the brain event-related potentials in children with Asperger syndrome. Clin Neurophysiol 117: 2161–2171. 16890012 10.1016/j.clinph.2006.06.709

[60] Stevenson CM, Brookes MJ, Morris PG (2011) beta-Band correlates of the fMRI BOLD response. Hum Brain Mapp 32: 182–197. 21229612 10.1002/hbm.21016 PMC6870318

[61] Yoncheva YN, Zevin JD, Maurer U, McCandliss BD (2010) Auditory selective attention to speech modulates activity in the visual word form area. Cereb Cortex 20: 622–632. 19571269 10.1093/cercor/bhp129 PMC2820701

[62] Rama P, Relander-Syrjanen K, Carlson S, Salonen O, Kujala T (2012) Attention and semantic processing during speech: an fMRI study. Brain Lang 122: 114–119. 22672735 10.1016/j.bandl.2012.04.018

[63] Branch C, Milner B, Rasmussen T (1964) Intracarotid Sodium Amytal for the Lateralization of Cerebral Speech Dominance; Observations in 123 Patients. J Neurosurg 21: 399–405. 14168208 10.3171/jns.1964.21.5.0399

